# The Cesspools of Chichester: A Courageous Speech

**Published:** 1889-11-23

**Authors:** R. Durnford

**Affiliations:** The Bishop of Chichester


### The Cesspools of Chichester: A Courageous Speech.

By the Bishop of Chichester, the Right Reverend R. Durnford, D.D.

We referred last week in our article "A City of Cesspools" to the unjustifiable silence of the clergy, seeing that the lives of their people were at stake. We are very glad to see the Bishop has become alive to the situation, and the convincing speech he delivered at the Mayor's banquet ought to lead to the abolition of cesspools and the adoption of a complete system of water supply without delay. We are quite sure, too, that the local practitioners would welcome the election of that eminently practical, conciliatory and sound sanitarian, Dr. Kelly, to the vacant post of medical officer of health to the city, if he can be persuaded to accept so difficult and laborious a post, as this must prove to be, for some time to come, at any rate. The Bishop said:

The prosperity of Chichester is as dear to the clergy as it is to all in this room, and I imagine that we all feel that the old Romans, those masters of the world, made a very good choice when, 2,000 years ago, they fixed upon this place as the site of the city. I say they made a very good choice, for it has great natural advantages. It is sheltered from the cold north wind by a chain of hills; it basks in the full rays of a real sun, such as perhaps, of all the counties of England, Sussex alone enjoys. Its climate is tempered still more by its proximity to that summer sea, which brings visitors from all England to recover their health upon its favoured shores. It lies in the midst of a most fertile and productive district, and it has a ready outlet on all sides to a most delightful and wholesome country. It has within itself means of recreation for the inhabitants such as very few cities in this land, save London, possess. It has, too, the great advantage that those hills, of which I have spoken, which shelter the city from the northern and eastern blasts, contain an abundant supply of water, so inexhaustible, that in the driest season we remember, it never showed the least sign of failure. That is a prodigious advantage; and no water can compete with it for crystal clearness and wholesome qualities. That you will generally concede.

Well, you will say that every inhabitant of the city ought to have the advantage of drinking water so pure, so wholesome, so necessary to life. Is it the fact that every inhabitant of the city does drink this water? Is it not the fact rather that the poorer classes—those who can least help themselves—those at the base of the pyramid, are compelled to drink water absolutely unwholesome, very often teeming with visible animalculæ, and indeed it is not necessary to use a microscope in order to see worms and unclean things floating on its surface? That is a sad, a melancholy fact. We know that zymotic disease, as it is called—that insidious disease which creeps here and there, and penetrates into the palace as surely as it penetrates into the cottage—is fostered by this bad, corrupt, poisonous water. I imagine that no medical man in this place will deny that fact. Well, then, I am sure that as Chichester is dear to all of you, as all here assembled have a common interest in the health of the city, as we feel for those that are dependent upon us, and especially for our poorer neighbours, we all ought to rise up as one man in order to get rid of this reproach, that Chichester, which ought to have the best water in the county, is content with the very worst supply that the county possesses. I believe that there are many wells in this city absolutely putrid, giving forth water unfit for the commonest domestic purposes, which stinks as it comes from the well; and I believe that there are many other wells where the water is more or less unwholesome, and which is not conducive to human health or strength. Indeed, I believe that there are not any perfectly wholesome wells to be found within the compass of this city; all are more or less dangerous to human life (Hear, hear, and no, no). I trust I am not exaggerating; I speak from strong conviction and observation, and whether popular or unpopular I believe it to be the truth (applause).

I am glad to hear that cheer, because twenty years ago I dared to say the same thing in this room, and I was met with cries of opposition and disbelief; cries as if I was entering upon a province which I was not entitled to touch. Now I see that there is a very general feeling of sympathy with what I have said. If that be the case there surely is a remedy. Surely it is possible for the Sanitary Authority of this city, acting under the powers of an Act which I, in a humble way, put in use in a populous part of another county—acting, I say, under the very large powers of the Nuisances Removal and Diseases Prevention Act—to seal up all the unwholesome wells in the city, and thus indirectly compel the use of wholesome water. You will say, perhaps, that I am a shareholder in the water works, and that I am advocating the use of wholesome water in order to increase my dividends. I hope no gentleman in this room will impute to me so base a motive; as far as I know my own heart I am free from it.

I think another thing is required. How come the wells to be so polluted? It is because the whole ground is saturated with sewage matter; for, from the time of the Romans to this day, there has been a constant leakage of impurity, and the wells must, therefore, be more or less contaminated. It is of no use my being bishop of the diocese, and having an independent position, if I do not speak as an independent man. Remember, I am just as much a citizen of Chichester as any of you; and, as I wish for its prosperity, I desire to promote the first element of prosperity—the health and material welfare of all its inhabitants.

To come to the cause of the pollution of the soil, it is the leakage of the cesspools. It is an unsavoury subject, but it must be met and stated openly, that the wells admit leakage from other sources, which renders the water unfit for human use. Surely the first remedy should be to provide that these innumerable cesspools should be carefully inspected, should be made impervious to rainwater, should admit nothing but that which they were designed to admit, and should be periodically, and with great scrupulousness, cleansed and emptied. These are, I say, unsavoury things to mention, but they are necessary things. It is nonsense to palter with half-truths, and it is unworthy to be afraid to state what the whole truth is. Let us face the thing, and, seeing the danger in which all are in, let us meet it like men. I am obliged to you for hearing me so patiently. It is a subject I have long had on my mind, and I am glad of the opportunity of expressing what I had to say before an audience so capable of appreciating my statements, and of acting, if it please them, upon my suggestions.

